# Supplementary material for: Development of Machine-Learned Interatomic Potentials to Predict Structure, Transport, and Reactivity in Platinum-Based Fuel Cells
Source: ACS Omega. 2026 Jun 5;11(24):35662–71. doi: 10.1021/acsomega.6c01745 (PMC13294967; doi:10.1021/acsomega.6c01745)
Supplement: Supplementary file 1 [file ao6c01745_si_001.pdf]

Supporting Information: Development of machine-learned interatomic potentials to predict structure, transport, and reactivity in platinum-based fuel cells

Kamron Fazel,[1, a)] Sam Brown,[2, a)] Jacob Clary,[3] Pritom Bose,[4] Nima Karimitari,[5] Amalie L. Frischknecht,[6] Ravishankar Sundararaman,[1, b)] and Derek Vigil-Fowler[3, c)]

[1)] *Materials Science & Engineering, Rensselaer Polytechnic Institute, Troy, NY 12180, USA*

[2)] *Department of Chemistry and Biochemistry, New Mexico State University, Las Cruces, NM 88003, USA*

[3)] *Materials, Chemical, and Computational Science Directorate, National Renewable Energy Laboratory, Golden, CO 80401, USA*

[4)] *Mechanical, Aerospace & Nuclear Engineering, Rensselaer Polytechnic Institute, Troy, NY 12180, USA*

[5)] *Department of Chemistry and Biochemistry, University of South Carolina, Columbia, SC 29208, USA*

[6)] *Center for Integrated Nanotechnologies, Sandia National Laboratories, Albuquerque, New Mexico 87185, USA*

[a)] These authors contributed equally to this work.

[b)] Electronic mail: sundar@rpi.edu

[c)] Electronic mail: derek.vigil-fowler@nrel.gov

The Supporting Information contains additional summaries of the system properties of the AIMD systems used in initial model training, details on the active learning approach, additional DFT computational details, additional summary histograms of the AIMD and our MLIP bond lengths and angles for the Pt-Nafion-water composite system, more detail on our proton tracking methodology, additional planar-averaged density profiles of the composite system, and additional reaction pathway results.

I. MACE TRAINING DATA

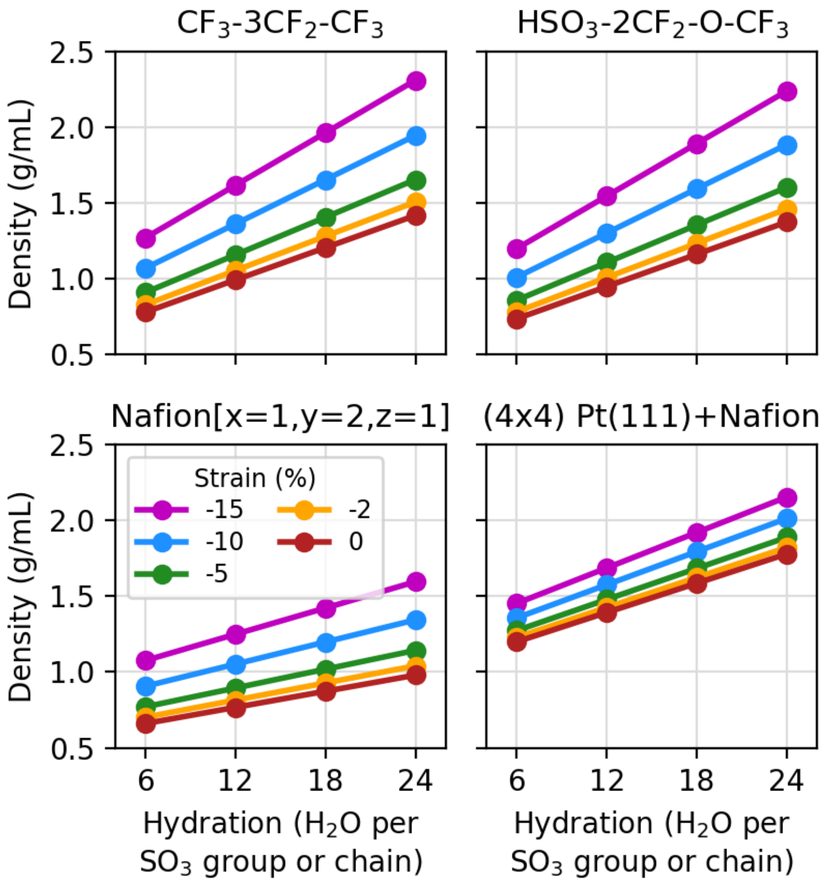

Figure S1: Densities of the Nafion+water region of all systems containing Nafion in the training set. For systems without Pt, the volume used is simply the total volume of the cell, V_{total}. For systems with Pt, the volume occupied by Nafion+water is defined as: $\mathrm{V} = \mathrm{V}_{\text{total}} - \mathrm{A}_{\text{xy}}\mathrm{H}_{\text{slab}}$, where H_{slab} is the center to center distance between top and bottom Pt rows plus double the covalent radius of a Pt atom.

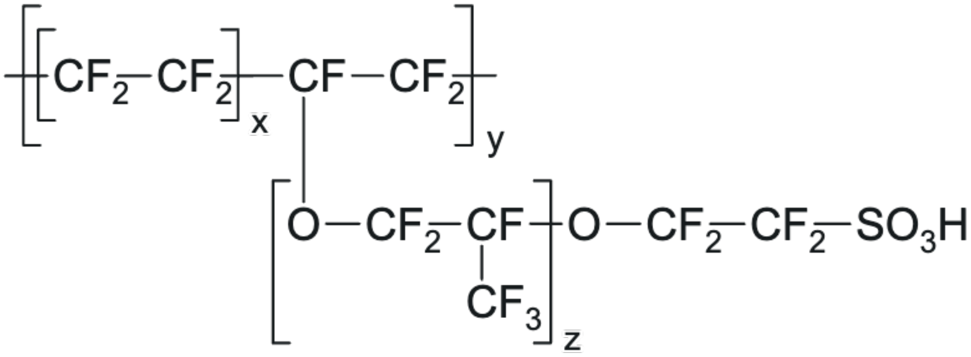

Figure S2: Nafion chemical structure used to generate AIMD water-Nafion and Pt-water-Nafion training data with x = 1, y = 2, and z = 1. An F atom was added to the terminating CF_2 groups along the backbone. Each AIMD-simulated system included 1 Nafion chain with a hydration level of 12 H_2O per SO_3 tail group. One SO_3 tail group was initialized deprotonated, with one water molecule correspondingly replaced by hydronium.

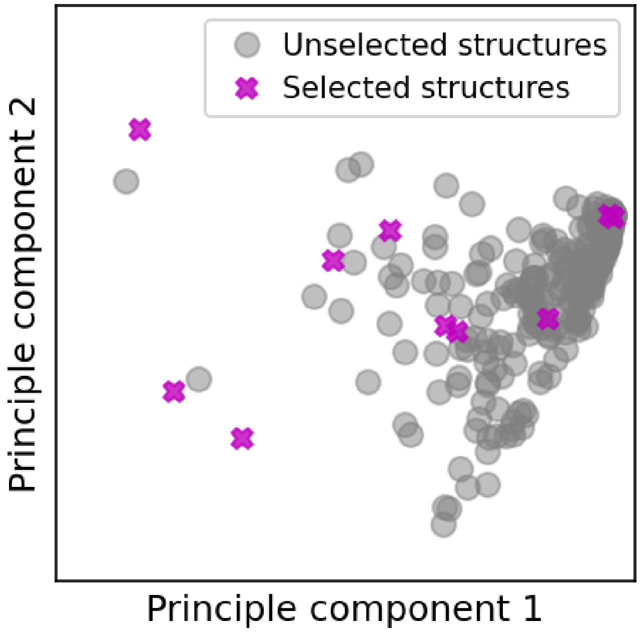

Figure S3: Principle component analysis plot for different Nafion[x=1,y=2,z=1] rotations over a (4×4) Pt(111) surface. The gray circles denote rotations there were not selected for AIMD runs while the magenta Xs denote the 10 rotations selected for AIMD runs with different hydration levels and strains.

II. MACE TRAINING

The following parameters were used to train the MACE models, with the only difference between the committee of models being the seed.

```
mace_run_train \
    --name="mace-r2-step10-300k" \
    --train_file="train.xyz" \
    --energy_key energy \
    --forces_key forces \
    --valid_fraction=0.03 \
    --config_type_weights='{"Default":1.0}' \
    --E0s="{1:-13.600891103331842, \
            6:-147.67494801444815, \
            8:-429.8144574591154, \
            9:-647.0870118967323, \
            16:-275.5108875572458, \
            78:-3295.2788163081627}" \
    --model="MACE" \
    --hidden_irreps='128x0e + 128x1o' \
    --r_max=5.0 \
    --batch_size=8 \
    --max_num_epochs=500 \
    --ema \
    --ema_decay=0.99 \
    --amsgrad \
    --distributed \
    --restart_latest \
    --save_all_checkpoints \
    --device=cuda \
    --seed=1337
```

III. MACE ACTIVE LEARNING

Equation S1 was used to determine the relative force deviation for each atom in the active learning process.

$$\sigma_{\text{relative}}(i) = \frac{\sigma(|F_i|)}{\langle|F_i|\rangle + 0.2} \tag{S1}$$

where $\sigma_{\text{relative}}(i)$ is the i-th particle's relative deviation, $\sigma(|F_i|)$ is the i-th particle's force magnitude standard deviation across the committee, and $\langle|F_i|\rangle$ is the i-th particle's average force magnitude across the committee. The additive 0.2 is to avoid division by a near zero average force magnitude.

During each active learning iteration, one of the three models was used to produce 24 separate 1 ps NVT trajectories, where each of the trajectories was initialized using the last snapshot of a corresponding training AIMD trajectory as the starting point. The 24 initial configurations used were water at both 0 and -5% strain, Nafion-water systems at -10% linear strain and both λ=12 and 18, and Pt-Nafion-water systems with all 10 polymer rotations at -10% linear strain and both λ=12 and 18. Following generation of each trajectory, the other two models were used to predict the forces and energies for all frames in each same trajectory. The frames with a maximum relative force magnitude deviation, calculated using equation SS1, between 0.0600 and 0.0625 across all 3 models were then flagged for DFT validation. The DFT calculations on these frames used the same settings as described above and were used to generate new forces and energies added to the training set for the next iteration of models.

Table S1: Initial iteration active learning flagged frames, average per-frame maximum relative force magnitude deviation, average per-frame relative force magnitude deviation, and average per-frame energy deviation per atom (meV/atom). Relative force deviations were calculated using equation S1. All Nafion and Pt-Nafion systems were run with -10% linear strain, while water was run with 0 and -5% strain. All systems were run at 300 K using Langevin dynamics.

**System**	**Rotation Index**	**Hydration**	**Flagged Frames**	$\langle \sigma_{\mathrm{rel,max}}(\lvert F \rvert) \rangle$	$\langle \sigma_{\mathrm{rel}}(\lvert F \rvert) \rangle$	$\langle \sigma(\lvert E \rvert) \rangle$
Water, 0% strain	–	–	0	0.0093	0.0021	0.211
Water, -5% strain	–	–	0	0.0099	0.0022	0.277
Nafion	–	12	0	0.0220	0.0045	1.775
Nafion	–	18	1	0.0200	0.0039	1.420
Pt+Nafion	1	12	7	0.0327	0.0065	0.187
Pt+Nafion	1	18	10	0.0345	0.0067	0.117
Pt+Nafion	2	12	11	0.0327	0.0065	0.173
Pt+Nafion	2	18	16	0.0366	0.0068	0.266
Pt+Nafion	3	12	108	0.0584	0.0091	0.340
Pt+Nafion	3	18	97	0.0790	0.0097	0.291
Pt+Nafion	4	12	107	0.0661	0.0094	0.178
Pt+Nafion	4	18	24	0.0409	0.0071	0.127
Pt+Nafion	5	12	2	0.0319	0.0067	0.243
Pt+Nafion	5	18	16	0.0367	0.0067	0.150
Pt+Nafion	6	12	30	0.0407	0.0074	0.181
Pt+Nafion	6	18	28	0.0428	0.0078	0.133
Pt+Nafion	7	12	14	0.0345	0.0066	0.198
Pt+Nafion	7	18	13	0.0369	0.0069	0.108
Pt+Nafion	8	12	16	0.0388	0.0074	0.178
Pt+Nafion	8	18	20	0.0370	0.0070	0.115
Pt+Nafion	9	12	8	0.0336	0.0068	0.166
Pt+Nafion	9	18	9	0.0364	0.0072	0.161
Pt+Nafion	10	12	66	0.0477	0.0080	0.179
Pt+Nafion	10	18	70	0.0501	0.0079	0.184
**Average**				**0.0379**	**0.0067**	**0.307**

Table S2: Iteration 1 active learning flagged frames, average per-frame maximum relative force magnitude deviation, average per-frame relative force magnitude deviation, and average per-frame energy deviation per atom (meV/atom). Relative force deviations were calculated using equation S1. All Nafion and Pt-Nafion systems were run with -10% linear strain, while water was run with 0 and -5% strain. All systems were run at 300 K using Langevin dynamics.

**System**	**Rotation Index**	**Hydration**	**Flagged Frames**	$\langle \sigma_{\mathrm{rel,max}}(\lvert F \rvert) \rangle$	$\langle \sigma_{\mathrm{rel}}(\lvert F \rvert) \rangle$	$\langle \sigma(\lvert E \rvert) \rangle$
Water, 0% strain	–	–	0	0.0098	0.0022	0.524
Water, -5% strain	–	–	0	0.0104	0.0023	0.369
Nafion	–	12	0	0.0230	0.0048	1.398
Nafion	–	18	0	0.0208	0.0040	1.140
Pt+Nafion	1	12	9	0.0318	0.0066	0.059
Pt+Nafion	1	18	9	0.0328	0.0065	0.079
Pt+Nafion	2	12	3	0.0307	0.0064	0.119
Pt+Nafion	2	18	8	0.0351	0.0066	0.101
Pt+Nafion	3	12	34	0.0449	0.0077	0.144
Pt+Nafion	3	18	67	0.0712	0.0090	0.090
Pt+Nafion	4	12	38	0.0444	0.0079	0.071
Pt+Nafion	4	18	14	0.0384	0.0072	0.060
Pt+Nafion	5	12	7	0.0313	0.0066	0.087
Pt+Nafion	5	18	8	0.0330	0.0066	0.126
Pt+Nafion	6	12	16	0.0379	0.0071	0.049
Pt+Nafion	6	18	28	0.0404	0.0074	0.052
Pt+Nafion	7	12	4	0.0322	0.0064	0.052
Pt+Nafion	7	18	11	0.0347	0.0067	0.097
Pt+Nafion	8	12	7	0.0362	0.0071	0.082
Pt+Nafion	8	18	7	0.0358	0.0069	0.097
Pt+Nafion	9	12	5	0.0343	0.0069	0.073
Pt+Nafion	9	18	15	0.0348	0.0069	0.109
Pt+Nafion	10	12	42	0.0438	0.0074	0.091
Pt+Nafion	10	18	55	0.0455	0.0074	0.083
**Average**				**0.0347**	**0.0064**	**0.215**

Table S3: Iteration 2 active learning flagged frames, average per-frame maximum relative force magnitude deviation, average per-frame relative force magnitude deviation, and average per-frame energy deviation per atom (meV/atom). Relative force deviations were calculated using equation S1. All Nafion and Pt-Nafion systems were run with -10% linear strain, while water was run with 0 and -5% strain. All systems were run at 300 K using Langevin dynamics.

**System**	**Rotation Index**	**Hydration**	$\langle \sigma_{\mathrm{rel,max}}(\lvert F \rvert) \rangle$	$\langle \sigma_{\mathrm{rel}}(\lvert F \rvert) \rangle$	$\langle \sigma(\lvert E \rvert) \rangle$
Water, 0% strain	–	–	0.0096	0.0022	1.220
Water, -5% strain	–	–	0.0102	0.0023	1.159
Nafion	–	12	0.0217	0.0045	0.521
Nafion	–	18	0.0199	0.0039	0.358
Pt+Nafion	1	12	0.0320	0.0065	0.115
Pt+Nafion	1	18	0.0354	0.0066	0.086
Pt+Nafion	2	12	0.0315	0.0064	0.112
Pt+Nafion	2	18	0.0340	0.0066	0.103
Pt+Nafion	3	12	0.0430	0.0075	0.116
Pt+Nafion	3	18	0.0567	0.0080	0.121
Pt+Nafion	4	12	0.0454	0.0079	0.086
Pt+Nafion	4	18	0.0386	0.0071	0.063
Pt+Nafion	5	12	0.0314	0.0066	0.109
Pt+Nafion	5	18	0.0319	0.0065	0.125
Pt+Nafion	6	12	0.0364	0.0069	0.109
Pt+Nafion	6	18	0.0398	0.0073	0.070
Pt+Nafion	7	12	0.0320	0.0066	0.121
Pt+Nafion	7	18	0.0370	0.0069	0.089
Pt+Nafion	8	12	0.0355	0.0071	0.108
Pt+Nafion	8	18	0.0343	0.0068	0.085
Pt+Nafion	9	12	0.0348	0.0068	0.104
Pt+Nafion	9	18	0.0363	0.0071	0.086
Pt+Nafion	10	12	0.0407	0.0071	0.118
Pt+Nafion	10	18	0.0434	0.0071	0.106
**Average**			**0.0338**	**0.0063**	**0.220**

Table S4: Energy (meV/atom) and force (meV/Å) evaluation error of our trained MACE models evaluated on a common test set.

**Iteration**	$\langle \mathrm{RMSE}(E) \rangle$	$\langle \mathrm{RMSE}(F) \rangle$
Preliminary	3.50	49.17
Iteration 1	3.43	49.23
Iteration 2	3.90	49.33

Table S5: Ablation test energy (meV/atom) and force (meV/Å) error with varying training dataset compositions. The first row shows the errors for the full dataset, while subsequent rows show the errors when we removed the part of the full dataset indicated in the "Removal from Full Training Dataset" column, e.g. row 4 gives the errors when we removed the training data of the Nafion water systems.

**Removal from Full Training Dataset**	$\langle \text{RMSE}(E) \rangle$	$\langle \text{RMSE}(F) \rangle$
None	3.5	49.17
Half of Nafion Rotations on Platinum	3.4	63.4
Strained systems	8.0	189.7
Nafion-water	11.3	59.5
Strained systems except -15%	4.2	60.1

IV. ADDITIONAL DFT COMPUTATIONAL DETAILS

The Pt surface was built by expanding the fcc Pt bulk primitive cell into a (4×4) supercell with 4 layers and 64 total atoms. We used a 2×2×1 Γ-centered **k**-grid and 0.2 eV Fermi electronic smearing for all systems containing the (4×4) Pt(111) surface and a 1×1×1 Γ-centered **k**-grid for systems not containing Pt. Each AIMD trajectory in the training data set was generated using the NVT ensemble at 300 K using a Nose-Hoover thermostat with 1 fs timesteps. The wavefunctions were converged to an energy threshold of 10^{-6} hartrees at each time step.

V. DETAILS OF THE CLASSICAL MD SIMULATIONS

Temperature and pressure were controlled with a Nose-Hoover thermostat and barostat with time constants of 0.1 ps and 1 ps, respectively. A short range cutoff of 10 Å was used for intermolecular interactions, while long-range Coulomb interactions were computed using the particle-particle particle-mesh (PPPM) algorithm with an accuracy of 10^{-5}.

For the Pt-Nafion-water systems, a platinum slab with 4 atomic layers was built using the same procedure as for the DFT calculations, and had lateral dimensions of $L_x = 33.2$ Å and $L_y = 38.3$ Å. The Pt surface and the Nafion-water mixture as built by EMC were combined in LAMMPS. The interactions between the Pt atoms and other atoms in the system were obtained from Brunello, et al.[1] The Pt atoms were held fixed throughout the simulations. The thermostat was applied only to the polymer, water, and hydronium ions. The initial system was first compressed perpendicular to the Pt surfaced to $L_z = 65$ Å, and subsequent

simulations applied the barostat only in the z-direction, keeping the lateral dimensions fixed throughout. Periodic boundary conditions were applied in all directions, so there are two Pt-polymer interfaces in the system.

Both the bulk and composite systems were subjected to an annealing protocol involving successive cycles of MD simulations at high temperatures and pressures in order to overcome large energy barriers. In the initial annealing runs, the atomic mass of the F atoms was set to 2.0 g/mol to help speed the polymer dynamics. Each cycle consisted of three MD runs: 1) a 50 ps NVT run at 1000 K, 2) a 100 ps NVT run at 300 K, and 3) a 50 ps NPT run at 300 K. A total of nine cycles were performed, progressively increasing the pressure to a high pressure and then decreasing back to 1 atm, using pressure values of 100, 1000, 10000, 5000, 1000, 500, 100, 10 and 1 atm. Following these cycles, the systems were equilibrated further in the NPT ensemble at 300 K and 1 atm for 2 ns.

The bulk Nafion systems were then annealed further, with an NPT run for 2 ns at 600K and 10 atm, then a ramp down to 353 K and 1 atm, at which point the F mass was reset to its correct value of 18.998 g/mol and the systems were run for another 2 ns. Finally, the systems were ramped down to 300 K and 1 atm and equilibrated for a final 1 ns. The total equilibration time for the bulk systems was approximately 8 ns. Final densities were 1.64, 1.60, and 1.59 g/cm^3, for the $\lambda = 9$, 12, and 15 Pt-Nafion-water systems, respectively. These densities are similar to those found in previous simulations of Nafion with the DREIDING force field and the SCP/Fw water model[2] and the densities of our Pt-Nafion-water training set (Figure S1). For the Pt-Nafion-water systems, after the annealing cycles the F mass was reset to 18.998 g/mol and the systems were further equilibrated at 300K and 1 atm for 1 ns, for a total equilibration time of approximately 3.5 ns.

VI. ADDITIONAL RDFS, BOND LENGTH HISTOGRAMS, AND BOND ANGLE HISTOGRAMS

Plotted below are AIMD and MACE MLIP MD results for predicted RDFs, and predicted bond length and bond angle histograms. As for bond lengths, Figures S9 and S10 show that our model is also in good agreement with AIMD for bond angle predictions. Our model and the AIMD bond angle histograms have closely aligned peak positions, with our model predicting sharper histograms for C-S-O, O-C-C, O-S-O, C-C-C, C-C-S, and C-O-C bond

angles, but broader histograms for H-O-H and O-S-O bond angles.

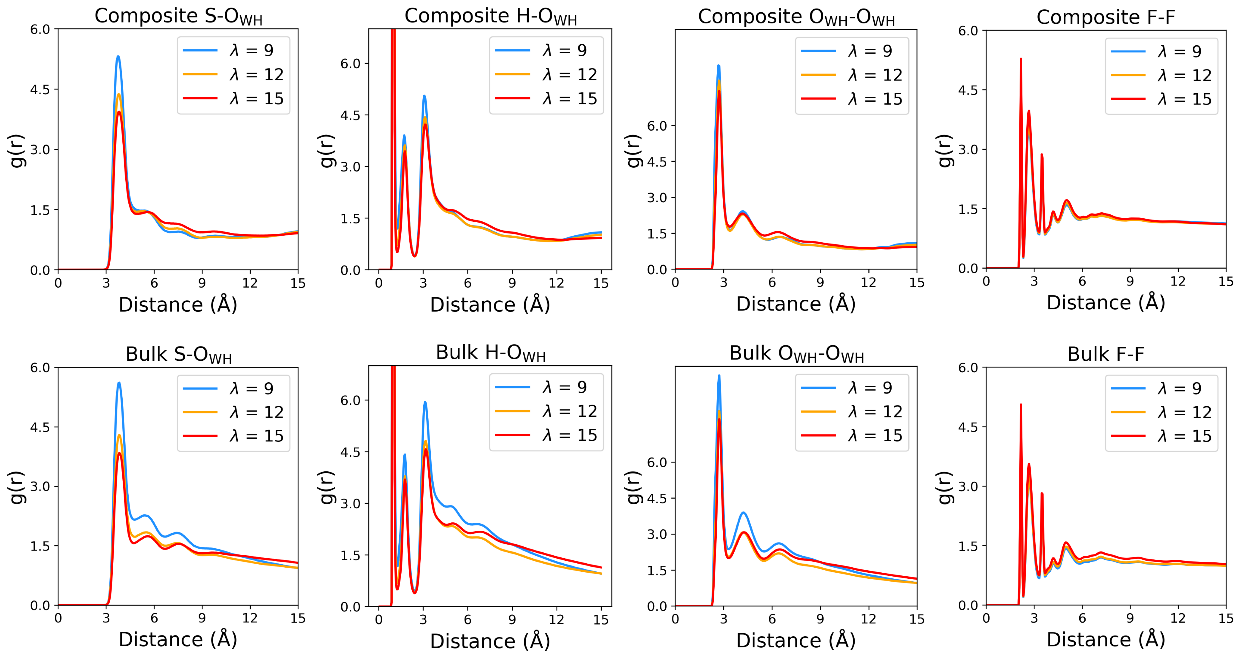

Figure S4: RDFs of bulk and composite systems for 1 ns MD trajectories generated using our trained MACE model.

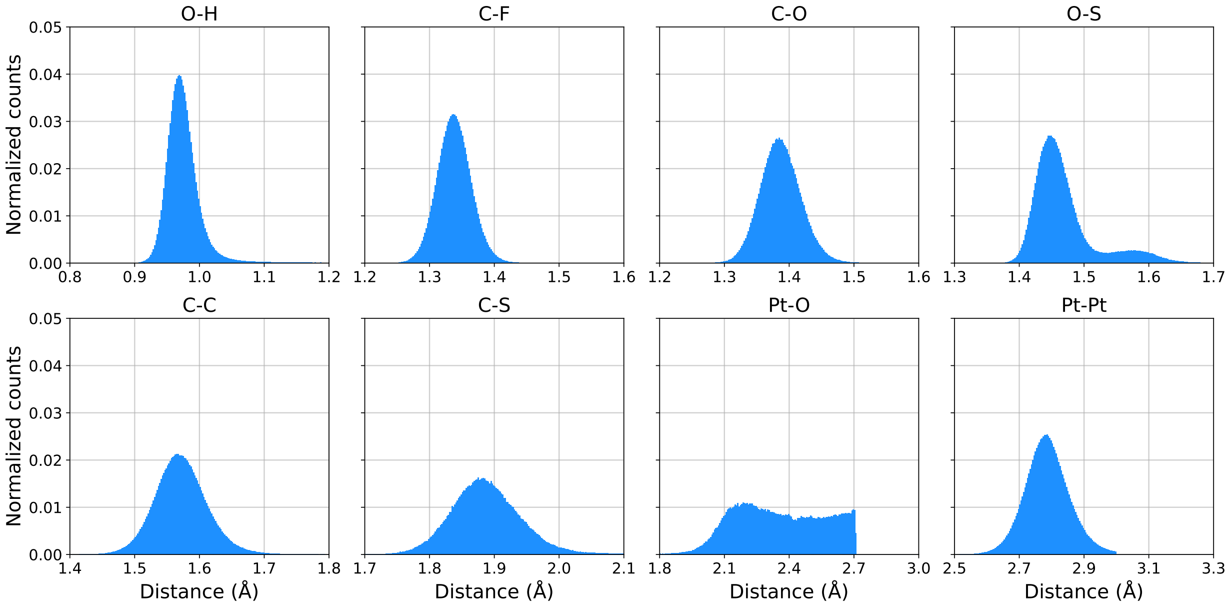

Figure S5: Bond distances of several atom type pairs extracted from the entire set of AIMD trajectories used to construct the training dataset. The subplots are sorted by mean bonding distance in ascending order. A bonded pair between atoms A_1 and A_2 was defined as occurring when the 2 atoms were closer than $\alpha(r_{A_1} + r_{A_2})$, where r_{A_1} and r_{A_2} are the covalent radii of A_1 and A_2. α is a scale factor tuned to be 1.341 so that the maximum distance for an O-H bond was 1.3 Å. For Pt-O, the scale factor results in a cutoff of 2.7 Å, resulting in a sharp distribution cutoff as oxygen atoms are present in water far from the surface. We note that the second small peak near 1.6 Å for the AIMD O-S histogram occurs when a sulfonate oxygen atom is protonated. This peak is only present for AIMD trajectories because sulfonate groups were initialized in their protonated state. In contrast, starting structures for our MLIP trajectories were initialized with deprotonated sulfonate groups and hydronium.

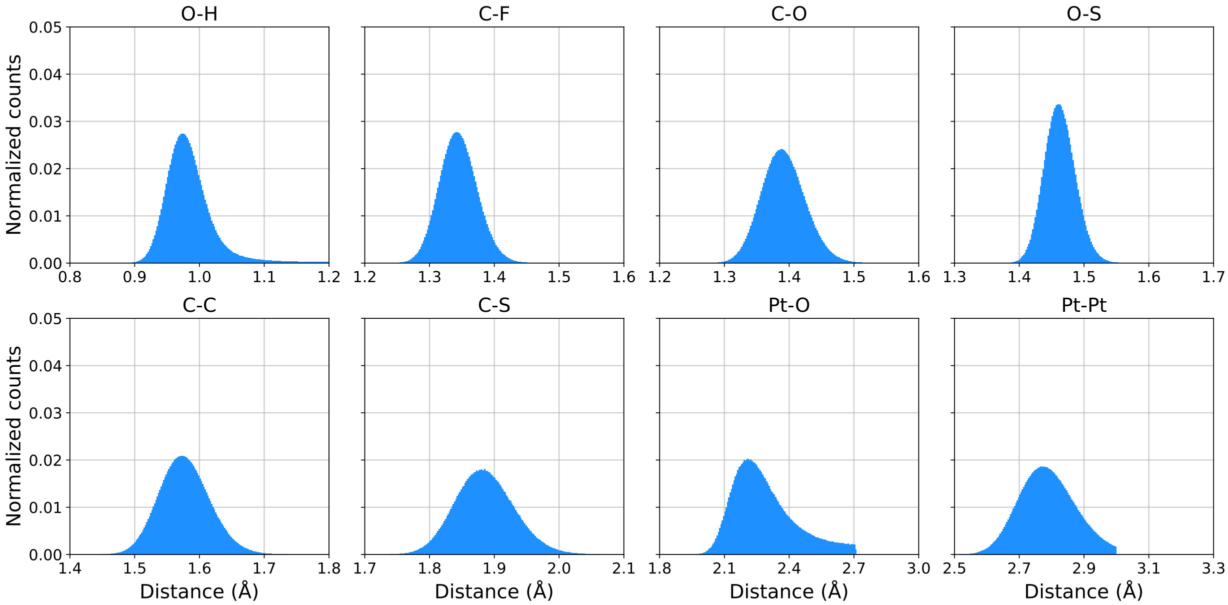

Figure S6: Bond distances of several atom type pairs across both bulk and composite systems extracted from 1 ns MD trajectories generated using our trained MACE model. The subplots are sorted by mean bonding distance in ascending order. A bonded pair between atoms A_1 and A_2 was defined as occurring when the 2 atoms were closer than $\alpha(r_{A_1} + r_{A_2})$, where r_{A_1} and r_{A_2} are the covalent radii of A_1 and A_2. α is a scale factor tuned to be 1.341 so that the maximum distance for an O-H bond was 1.3 Å. For Pt-O, the scale factor results in a cutoff of 2.7 Å, resulting in a sharp distribution cutoff as oxygen atoms are present in water far from the surface. We note that the second small peak near 1.6 Å for the AIMD O-S histogram occurs when a sulfonate oxygen atom is protonated. This peak is only present for AIMD trajectories because sulfonate groups were initialized in their protonated state. In contrast, starting structures for our MLIP trajectories were initialized with deprotonated sulfonate groups and hydronium.

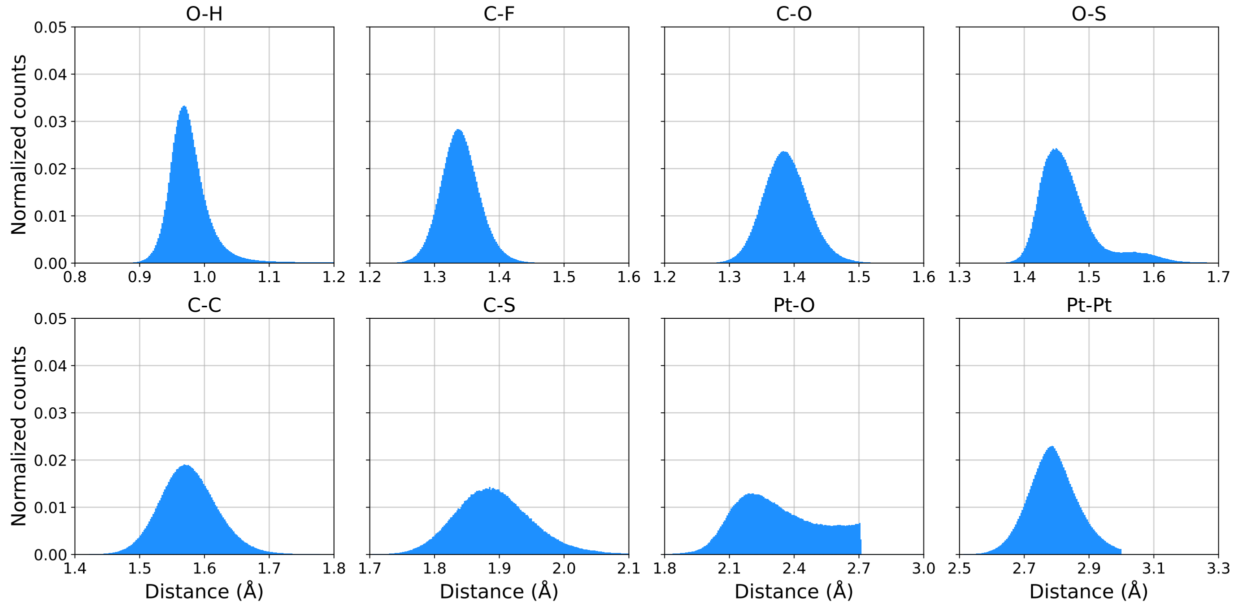

Figure S7: Bond distances of several atom type pairs extracted from 2 ps MD trajectories run on AIMD simulation cells with our trained MACE model with the same input structure as AIMD. The subplots are sorted by mean bonding distance in ascending order. A bonded pair between atoms A_1 and A_2 was defined as occurring when the 2 atoms were closer than $\alpha(r_{A_1} + r_{A_2})$, where r_{A_1} and r_{A_2} are the covalent radii of A_1 and A_2. α is a scale factor tuned to be 1.341 so that the maximum distance for an O-H bond was 1.3 Å. For Pt-O, the scale factor results in a cutoff of 2.7 Å, resulting in a sharp distribution cutoff as oxygen atoms are present in water far from the surface. We note that the second small peak near 1.6 Å for the AIMD O-S histogram occurs when a sulfonate oxygen atom is protonated. This peak is only present for AIMD trajectories because sulfonate groups were initialized in their protonated state. In contrast, starting structures for our MLIP trajectories were initialized with deprotonated sulfonate groups and hydronium.

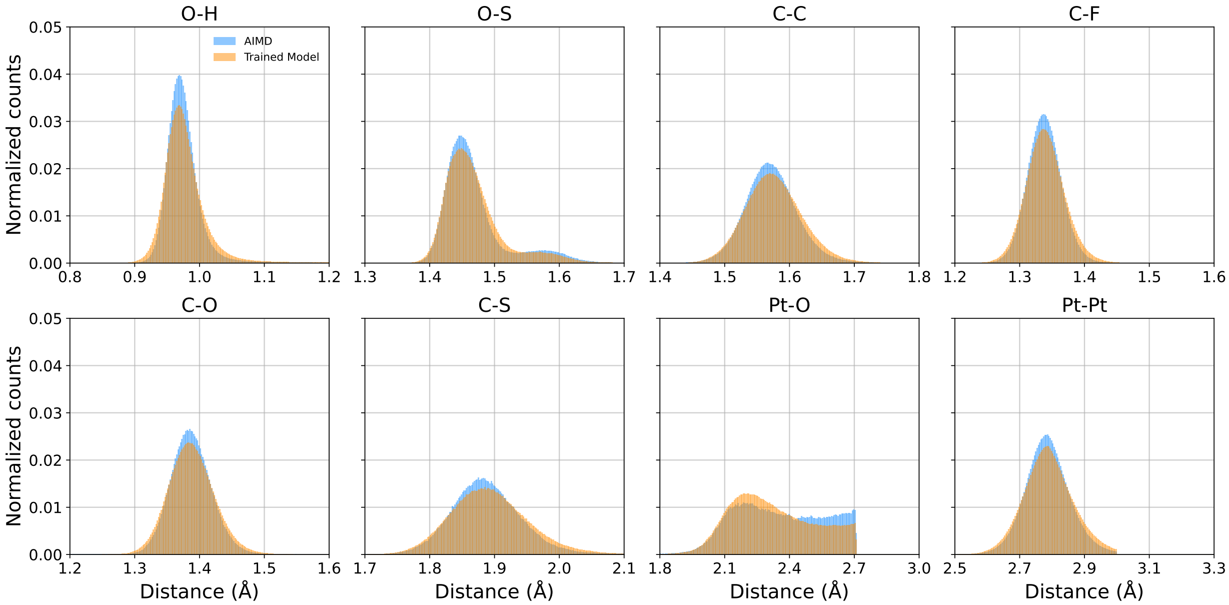

Figure S8: Overlayed bond distance of several atom type pairs extracted from 2 ps MD trajectories run on AIMD simulation cells with our trained MACE model with the same input structure as AIMD and the corresponding AIMD trajectory. The subplots are sorted by mean bonding distance in ascending order. A bonded pair between atoms A_1 and A_2 was defined as occurring when the 2 atoms were closer than $\alpha(r_{A_1} + r_{A_2})$, where r_{A_1} and r_{A_2} are the covalent radii of A_1 and A_2. α is a scale factor tuned to be 1.341 so that the maximum distance for an O-H bond was 1.3 Å. For Pt-O, the scale factor results in a cutoff of 2.7 Å, resulting in a sharp distribution cutoff as oxygen atoms are present in water far from the surface. We note that the second small peak near 1.6 Å for the AIMD O-S histogram occurs when a sulfonate oxygen atom is protonated. This peak is only present for AIMD trajectories because sulfonate groups were initialized in their protonated state. In contrast, starting structures for our MLIP trajectories were initialized with deprotonated sulfonate groups and hydronium.

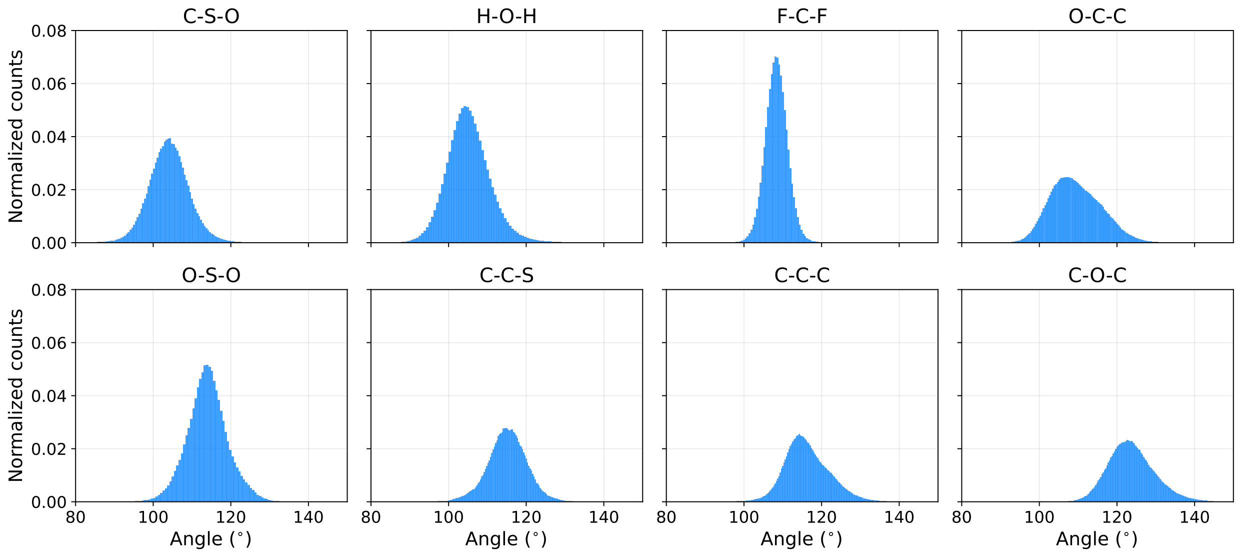

Figure S9: Bond angles of several atom type triplets extracted from the entire set of AIMD trajectories used to construct the training dataset. The subplots are sorted by mean angle magnitude in ascending order.

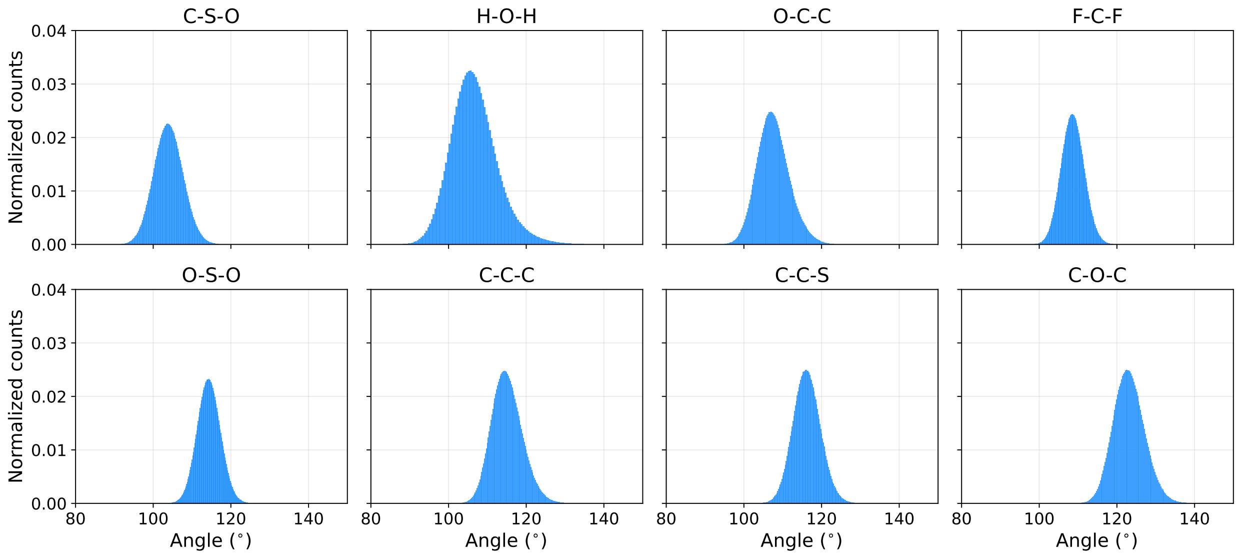

Figure S10: Bond angles of several atom type triplets across both bulk and composite systems extracted from 1 ns MD trajectories generated using our trained MACE model. The subplots are sorted by mean angle magnitude in ascending order.

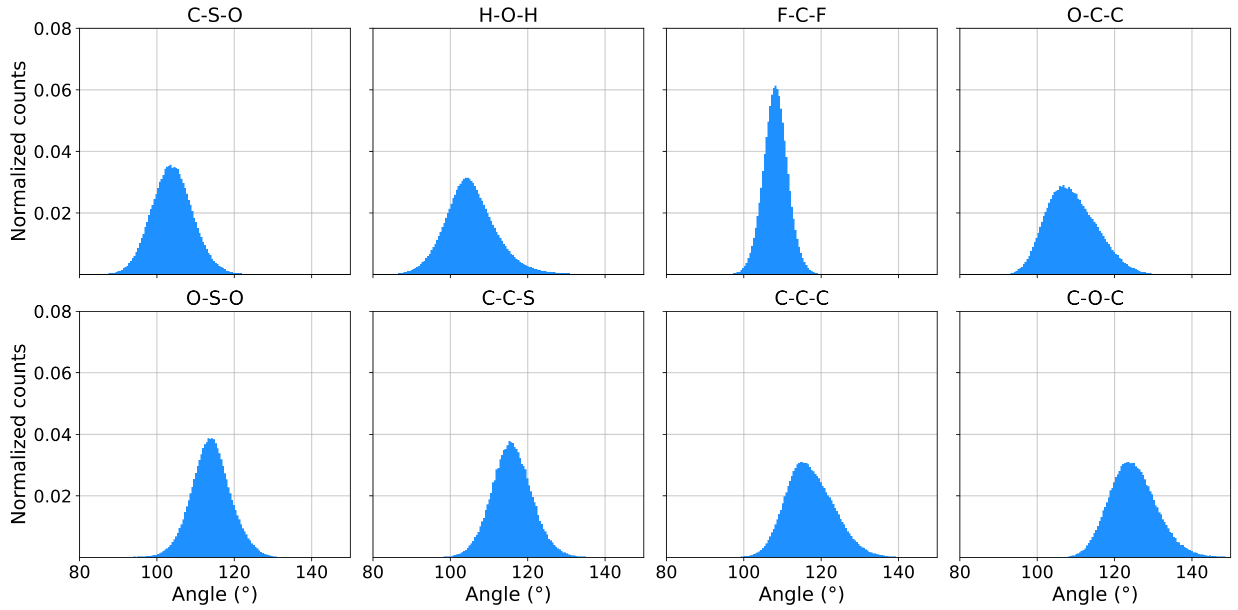

Figure S11: Bond angles of several atom type triplets across both bulk and composite systems extracted from simulations of AIMD cells using our trained model. The subplots are sorted by mean angle magnitude in ascending order.

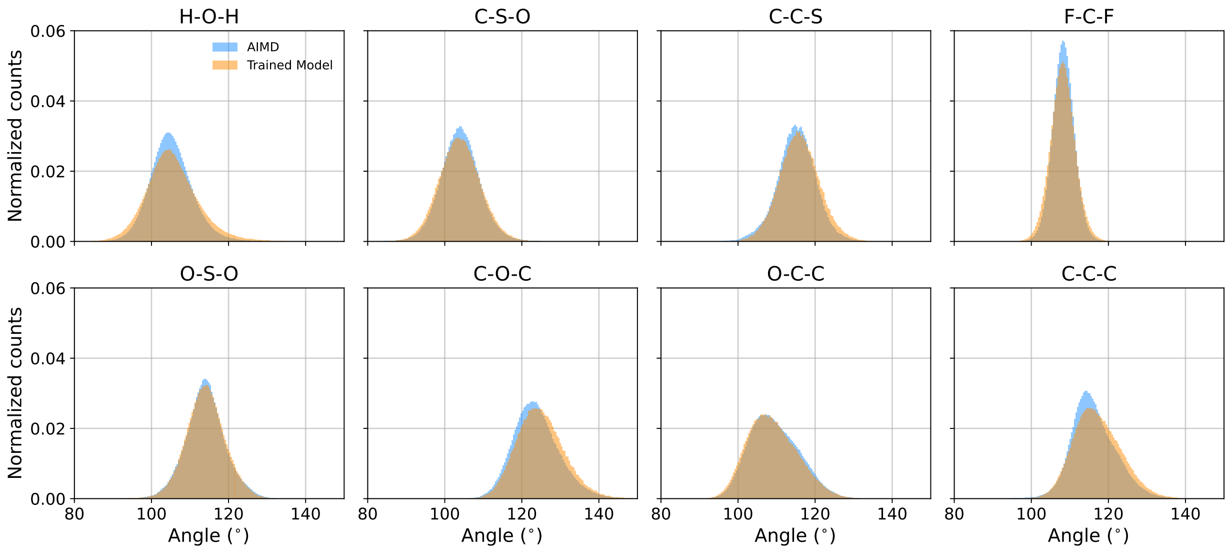

Figure S12: Bond angles of several atom type triplets across both bulk and composite systems extracted from simulations of AIMD cells using our trained model overlayed with AIMD. The subplots are sorted by mean angle magnitude in ascending order.

VII. PROTON TRACKING METHODOLOGY

The center of charge approach was developed to follow the positively charged particles of the system. In the bulk and composite systems of the present study, all positive charge starts as hydronium, and the oxygen position is tracked accordingly. At each evaluated frame in the trajectory, each positively charged particle is evaluated. If the hydronium continues as hydronium, the oxygen's coordinate is still tracked. Alternatively, if the hydronium loses a proton, the proton is tracked individually if it is free, or the newly formed hydronium if the proton hopped to a water molecule. In the case of cascades, a chain series of proton hopping events resulting in a large charge displacement, the proton defect is recursively traced to determine the positively charged particle, which is then tracked. Finally, in the case of complex formation, such as Zundel and Eigen ions, the acidic proton is determined as the shared proton for Zundel, or the smallest H_3O subparticle for Eigen ions. This methodology allows the MSD of the displaced charge to be determined, gathering insight into the Grotthuss hopping mechanism. After the coordinates are gathered, the MSD is determined through a time-lagged approach utilizing autocorrelation through the Wiener–Khinchin theorem[3].

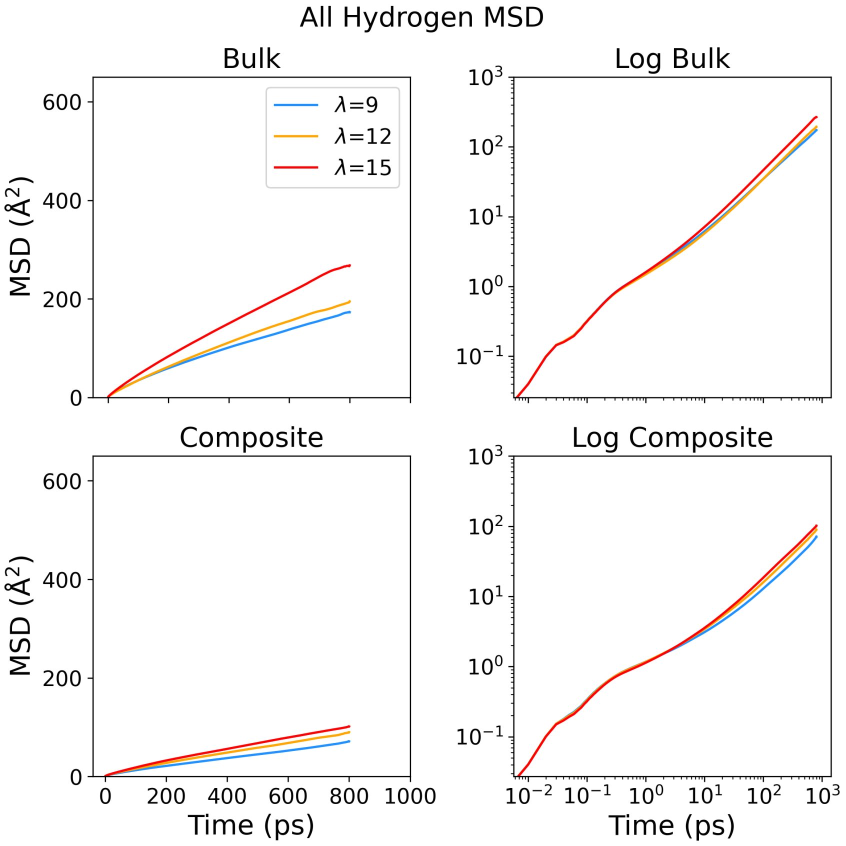

Figure S13: MSDs for all hydrogens in the system, calculated with MDAnalysis[4].

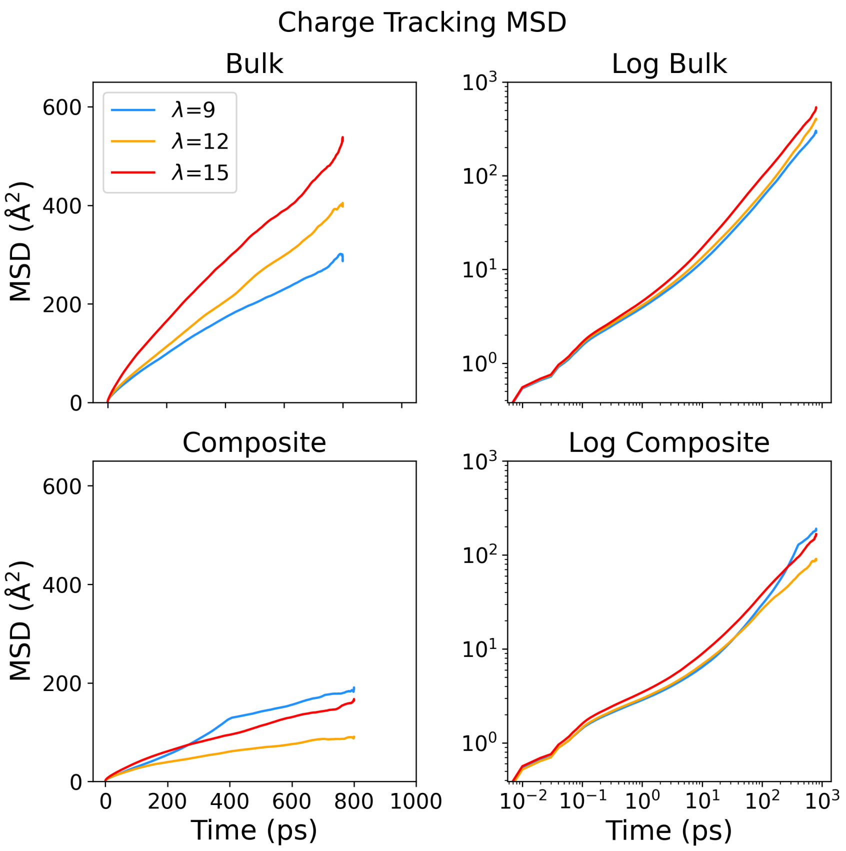

Figure S14: MSDs of proton defects calculated using the charged tracking methodology described in this section.

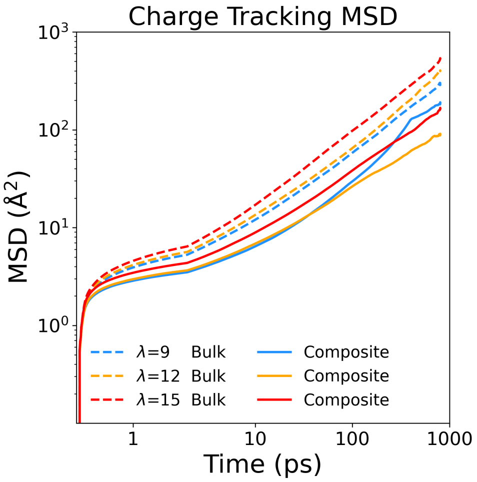

Figure S15: Log MSDs of proton defects calculated using the charged tracking methodology described in this section.

Table S6: MSDs (Å^2) at t = 600 ps for the MD trajectories generated using our MACE MLIP. The Bulk systems contain Nafion-water while the Composite systems contain Pt-Nafion-water.

λ	All Hydrogen		Charge Tracking	
	Bulk	Composite	Bulk	Composite
9	137.92	52.67	229.61	155.77
12	155.07	68.11	297.80	76.01
15	212.71	79.24	389.50	130.67

VIII. ADDITIONAL Z-AVERAGED DENSITY PROFILES

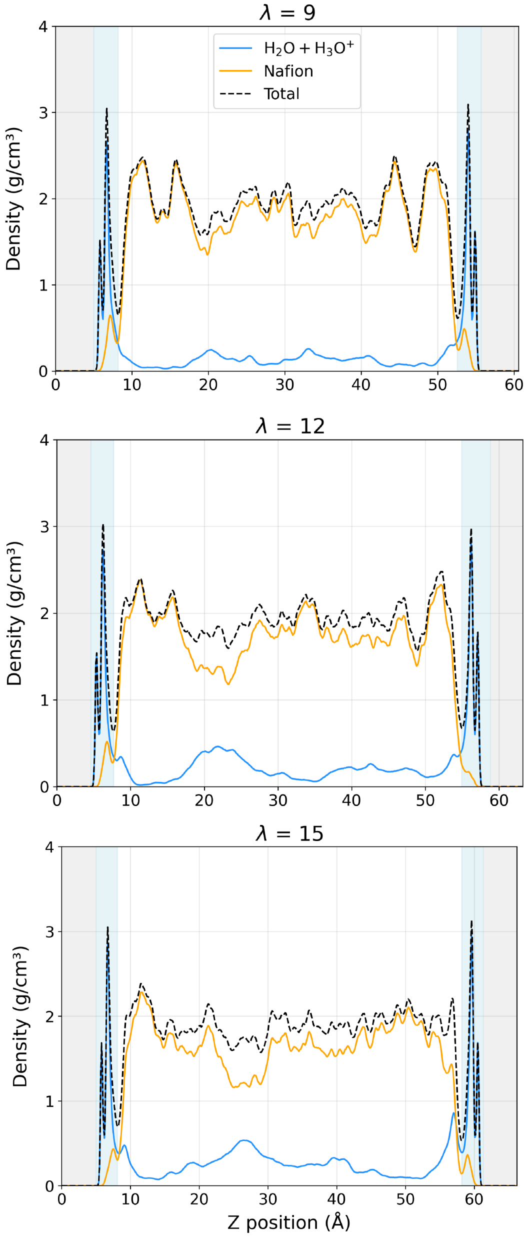

Figure S16: Planar-averaged density along the z-axis for the Pt-Nafion-water systems at 3 hydrations. The gray shaded region represents the platinum in the system, while the blue shaded region represents the surface water coordination region.

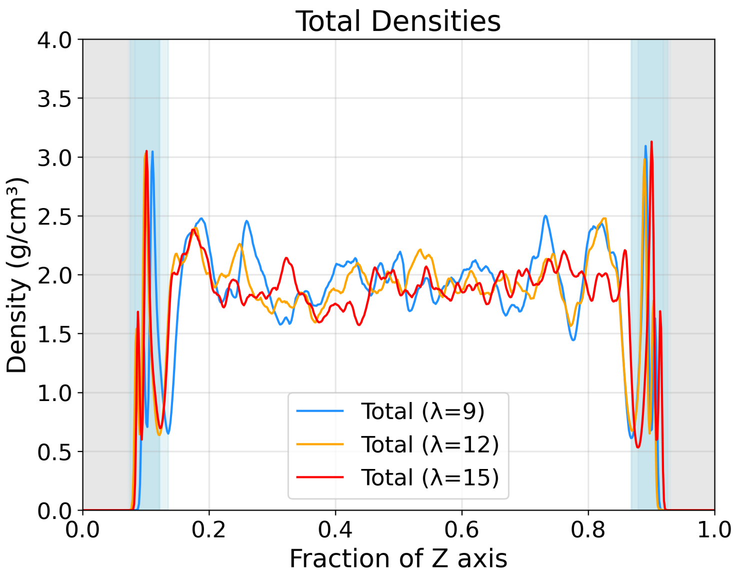

Figure S17: Total planar-averaged density along the z-axis of the Pt-Nafion-water systems at 3 hydrations. The z-axis for the 3 systems has been normalized to 1 to facilitate comparisons between hydration levels, which have different z axis lengths.

IX. ADDITIONAL REACTION PATHWAY RESULTS

These reaction pathways were generated by selecting a single structure from the end of a Pt-Nafion-water AIMD trajectory (λ=12 with no strain applied) included in the training set to use as the reactant state, transferring atoms along a reaction coordinate according to each reaction in the set, and then using r2SCAN to relax only the atoms involved in the reaction in both the initial or product state. This isolates the impact of the reaction on each total system energy as compared to a full ionic relaxation. The reaction intermediate images were generated from the relaxed endpoint images using the image-dependent pair potential method, as implemented in the Atomic Simulation Environment software package[5]. Intermediate image MLIP, PBE, or r2SCAN energies were then calculated without further ion optimization to provide benchmark energies, meaning that the structures and energies for these elementary steps are very likely not the actual transition states for each reaction. Given that the purpose of these calculations is to assess the accuracy of the MLIP in describing stretched-bond configurations relative to the functional used to generate their training sets, exact transition state pathways are not needed. We note that some reactions tested, such as SO_3 and CF_3 group separation from the Nafion chain, did not have a transition state in the pathway, and thus required freezing the central C and S atom in the dissociated product

state to prevent these groups from relaxing back to the reactant state.

These reaction pathways were generated from a λ=12 Pt-Nafion-water AIMD structure with no strain applied as the reactant state. Atoms were then transferred along each reaction coordinate above to a product state. Only the atoms involved in this reaction were then relaxed using r2SCAN to isolate the impact this each reaction on total system energy. The reaction intermediate images were generated from the relaxed endpoint images using the image-dependent pair potential method, as implemented in the Atomic Simulation Environment software package[5]. Intermediate image MLIP, PBE, or r2SCAN energies were then calculated without further ion optimization to provide benchmark energies, meaning that the structures and energies for these elementary steps are very likely not the actual transition states for each reaction. Given that the purpose of these calculations is to assess the accuracy of the MLIP in describing stretched-bond configurations relative to the functional used to generate their training sets, exact transition state pathways are not needed.

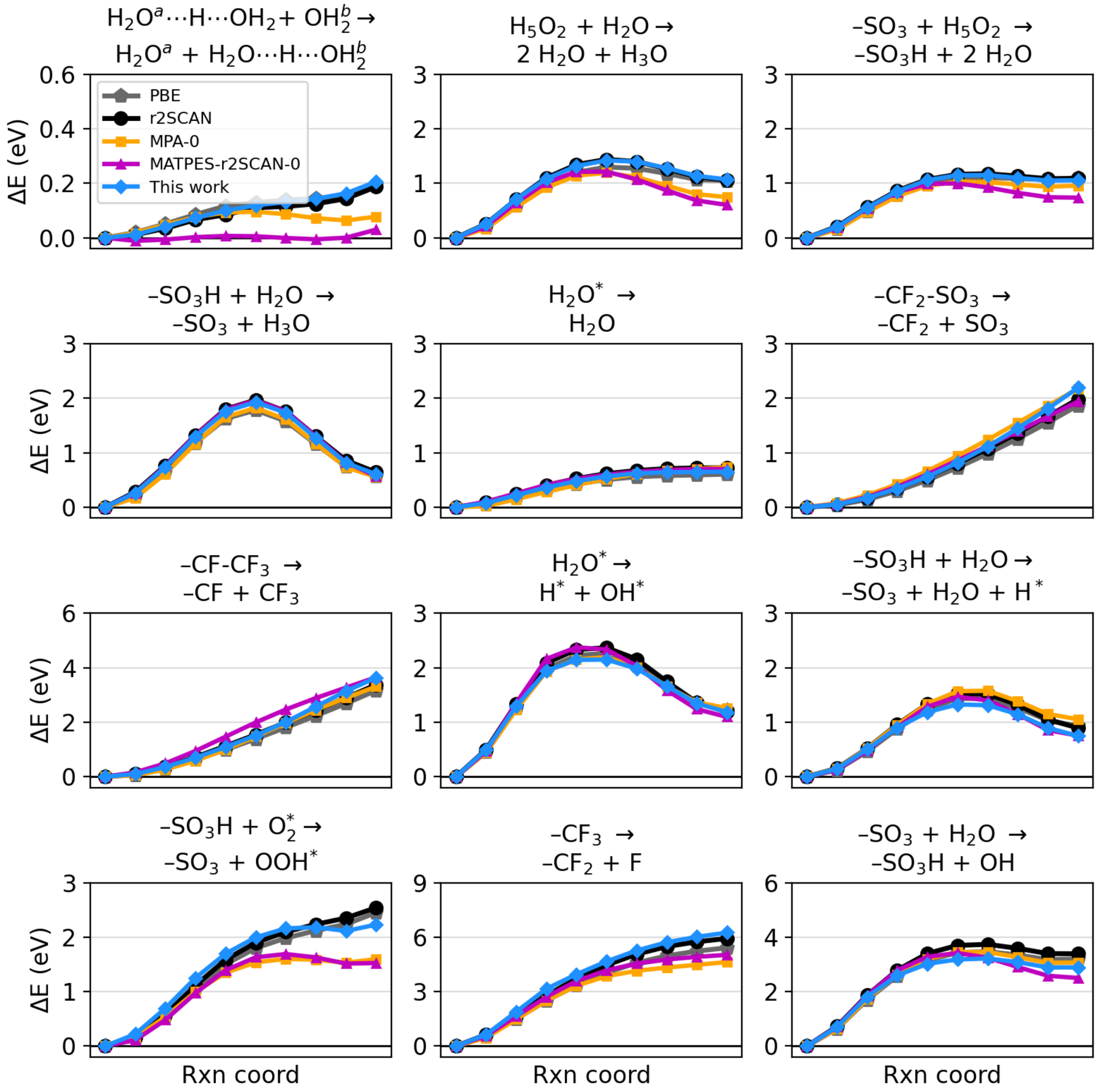

Figure S18: Reaction pathway energies predicted by the MLIP model trained in this work compared to those predicted by the MACE-MATPES-r2SCAN-0 medium model, MACE-MPA-0 medium model, and PBE and r2SCAN DFT calculations. We note that these pathways are single point energies for targeted atom transfers and are very likely not the true transition state energies for these elementary steps.

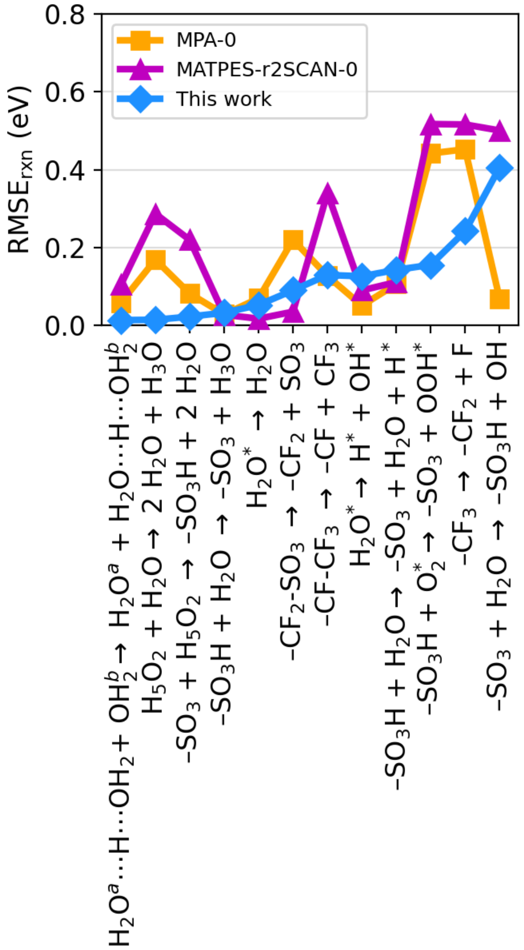

Figure S19: Reaction pathway energy root mean squared error (RMSE) averaged over all 10 images in each reaction pathway for the MLIP model trained in this work compared to the MACE-MATPES-r2SCAN-0 medium model, MACE-MPA-0 medium model, and r2SCAN DFT. The starting state (image 0) for each reaction was excluded from the RMSE calculations shown in this plot because its energy was always shifted to be 0 eV. We note that these pathways are single point energies for targeted atom transfers and are very likely not the true transition state energies for these elementary steps.

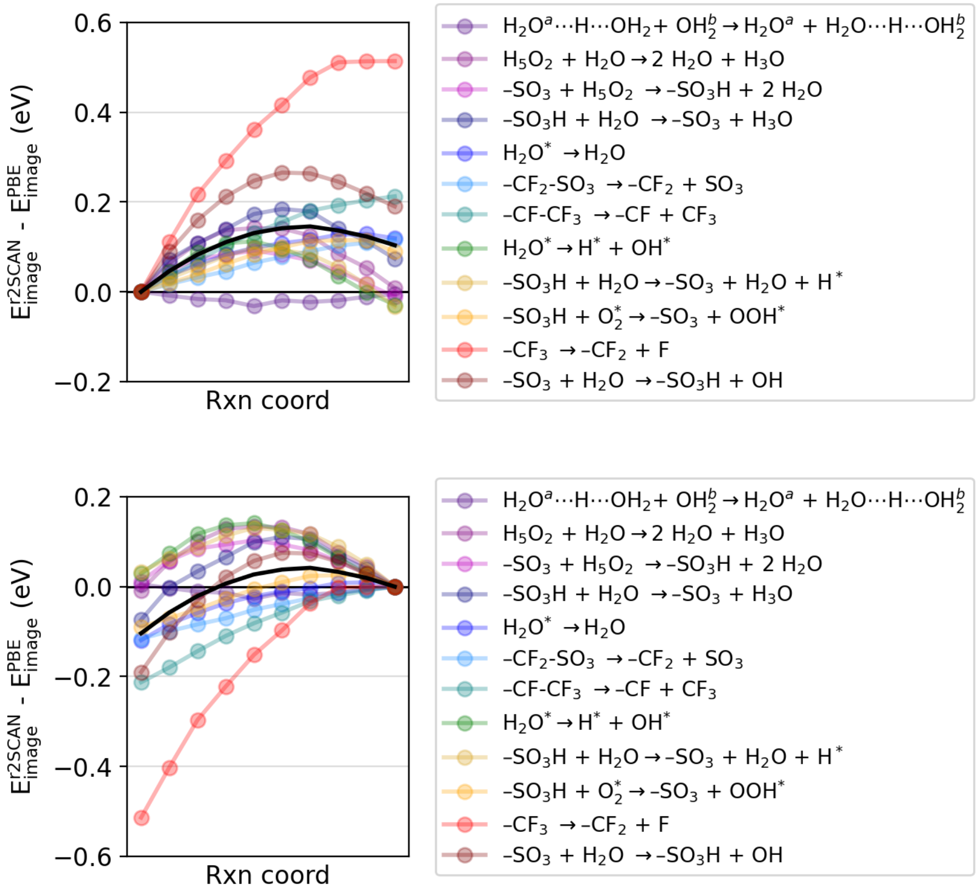

Figure S20: Reaction pathway energy differences for PBE vs r2SCAN. The average energy difference across all reactions at the same image index is shown as the solid black data. As the reaction coordinates of these pathways are arbitrary, the starting state (image 0) for each reaction was always shifted to be 0 eV in the top subplot, while the ending state (image 9) for each reaction was always shifted to be 0 eV in the bottom subplot. We note that these pathways are single point energies for targeted atom transfers and are very likely not the true transition state energies for these elementary steps.

REFERENCES

[1]G. F. Brunello, J. H. Lee, S. G. Lee, J. I. Choi, D. Harvey, and S. S. Jang, RSC Adv. **6**, 69670 (2016).

[2]Y.-L. S. Tse, A. M. Herring, K. Kim, and G. A. Voth, Journal Of Physical Chemistry C **117**, 8079 (2013).

[3]G. R. Kneller, V. Keiner, M. Kneller, and M. Schiller, Computer Physics Communications **91**, 191 (1995).

[4]R. Gowers, M. Linke, J. Barnoud, T. Reddy, M. Melo, S. Seyler, J. Domański, D. Dotson, S. Buchoux, I. Kenney, and O. Beckstein, in *PROC. OF THE 15th PYTHON IN SCIENCE CONF* (2016) pp. 98–105.

[5]A. H. Larsen, J. J. Mortensen, J. Blomqvist, I. E. Castelli, R. Christensen, M. Dułak, J. Friis, M. N. Groves, B. Hammer, C. Hargus, E. D. Hermes, P. C. Jennings, P. B. Jensen, J. Kermode, J. R. Kitchin, E. L. Kolsbjerg, J. Kubal, K. Kaasbjerg, S. Lysgaard, J. B. Maronsson, T. Maxson, T. Olsen, L. Pastewka, A. Peterson, C. Rostgaard, J. Schiøtz, O. Schütt, M. Strange, K. S. Thygesen, T. Vegge, L. Vilhelmsen, M. Walter, Z. Zeng, and K. W. Jacobsen, J. Phys.: Condens. Matter **29**, 273002 (2017).
